# Novel Utilization of a Penrose Drain as a Spacer During Temporary Artificial Urinary Sphincter Explantation and Rectourethral Fistula Repair

**DOI:** 10.7759/cureus.73137

**Published:** 2024-11-06

**Authors:** Jeremy Sheiber, Lucas R Wiegand

**Affiliations:** 1 Urology, Orlando Health/University of Central Florida College of Medicine, Orlando, USA; 2 Urology, Orlando Regional Medical Center, Orlando, USA

**Keywords:** artificial urinary sphincter, male urinary incontinence, post-prostatectomy incontinence, prostate cancer, radiation therapy complications

## Abstract

A 65-year-old male patient with a history of external beam radiation therapy for prostate cancer and multiple urological surgeries developed a rectourethral fistula after treatment for urethral diverticulum with stones. In managing this complex case, a Penrose drain was utilized as a spacer during artificial urinary sphincter cuff removal to preserve the urethral space for future sphincter re-implantation. This report highlights the novel application of a Penrose drain as a spacer in urological surgery and its benefits in minimizing tissue contraction and preserving urethral health.

## Introduction

Management of rectourethral fistulas (RUFs) in patients with a history of prostate cancer treatment, especially those who have undergone multiple urological reconstructions, presents significant challenges. RUFs, which are abnormal connections between the rectum and urethra, can occur as a result of varied prostate cancer treatments. The incidence is likely less than 1% with radiation therapy decreasing the chances of fistula repair success. Fistula repair rates are high in experienced centers - greater than 85%. Incontinence remains a risk after RUF repair with ablative therapies increasing risk for AUS placement [1]. These fistulas are associated with substantial morbidity, including urinary incontinence, fecaluria, and recurrent urinary tract infections, underscoring the complexity and necessity of effective repair [2].

The use of artificial urinary sphincters (AUSs) has been the gold standard for managing urinary incontinence since 1972. However, AUS use is not without complications, such as an increased risk of RUF and urethral erosion. In managing RUFs in patients with AUSs, particularly those treated for prostate cancer, preserving urethral integrity during healing is crucial to avoid complications like peri-urethral fibrosis or stricture formation, which can hinder future AUS reimplantation. While AUS use effectively controls urinary incontinence, it can complicate fistula repair due to a heightened risk of tissue erosion, especially in those with prior radiation therapy, as radiation reduces blood supply and impairs urethral healing [3,4].

For secondary AUS reimplantation, erosion rates can reach up to 35%, posing additional management challenges. Among those experiencing cuff erosion, up to 35% may ultimately require urinary diversion, such as suprapubic tube placement and urethral ligation, which significantly impacts quality of life [5]. Studies on AUS erosion and removal underscore the importance of careful planning to maintain urethral structure, as delayed healing or inadequate patency can hinder successful future interventions. Techniques such as using 10 French surgical drains or cuff sizers as spacers or selecting approaches like transcorporal cuff placement have been suggested for high-risk patients to allow space for healing without placing undue pressure on the urethral tissue [6-8]. It should be noted that such descriptions have been anecdotal, and no known peer-reviewed literature has been published on these outcomes to date [9,10].

In this case, a novel technique was employed in which a 1-inch Penrose drain was left in place as a temporary spacer following AUS cuff removal. The use of a Penrose drain in this context is rare but effective, providing a simple and flexible means of preserving the urethral lumen during fistula repair. By preventing tissue contraction and maintaining the urethral space, the drain facilitated successful healing and subsequent AUS reimplantation. This report highlights the effectiveness of this technique in preserving urethral patency during the healing period, enabling successful fistula repair and AUS replacement.

## Case presentation

The patient presented with a long history of urological complications. In 2002, he underwent external beam radiation therapy for prostate cancer. By 2017, the patient had developed a membranous urethral stricture, which failed to respond to multiple dilations over the prior several years. The patient then presented to the senior author and underwent posterior urethroplasty (excision and primary anastomosis) with a gracilis flap. He healed well from this with excellent urinary flow but did develop stress urinary incontinence, as expected. After a six-month period of healing and cystoscopic confirmation of a patent urethra, placement of a 4 cm cuff bulbar AUS was performed to manage urinary incontinence. The device was activated eight weeks later and the patient was able to manage with only a safety pad. Six months after the AUS placement, he underwent placement of an inflatable penile prosthesis via a suprapubic approach.

By 2023, the patient had developed a diverticulum at the site of anastomosis, complicated by stones. Following laser cystolitholapaxy for stone extraction, the patient developed an RUF despite deactivating the AUS to reduce pressure on the urethra. Prolonged deactivation did not resolve the fistula. The patient underwent 60 sessions of hyperbaric oxygen therapy. He had no fecal incontinence.

**Figure 1:**
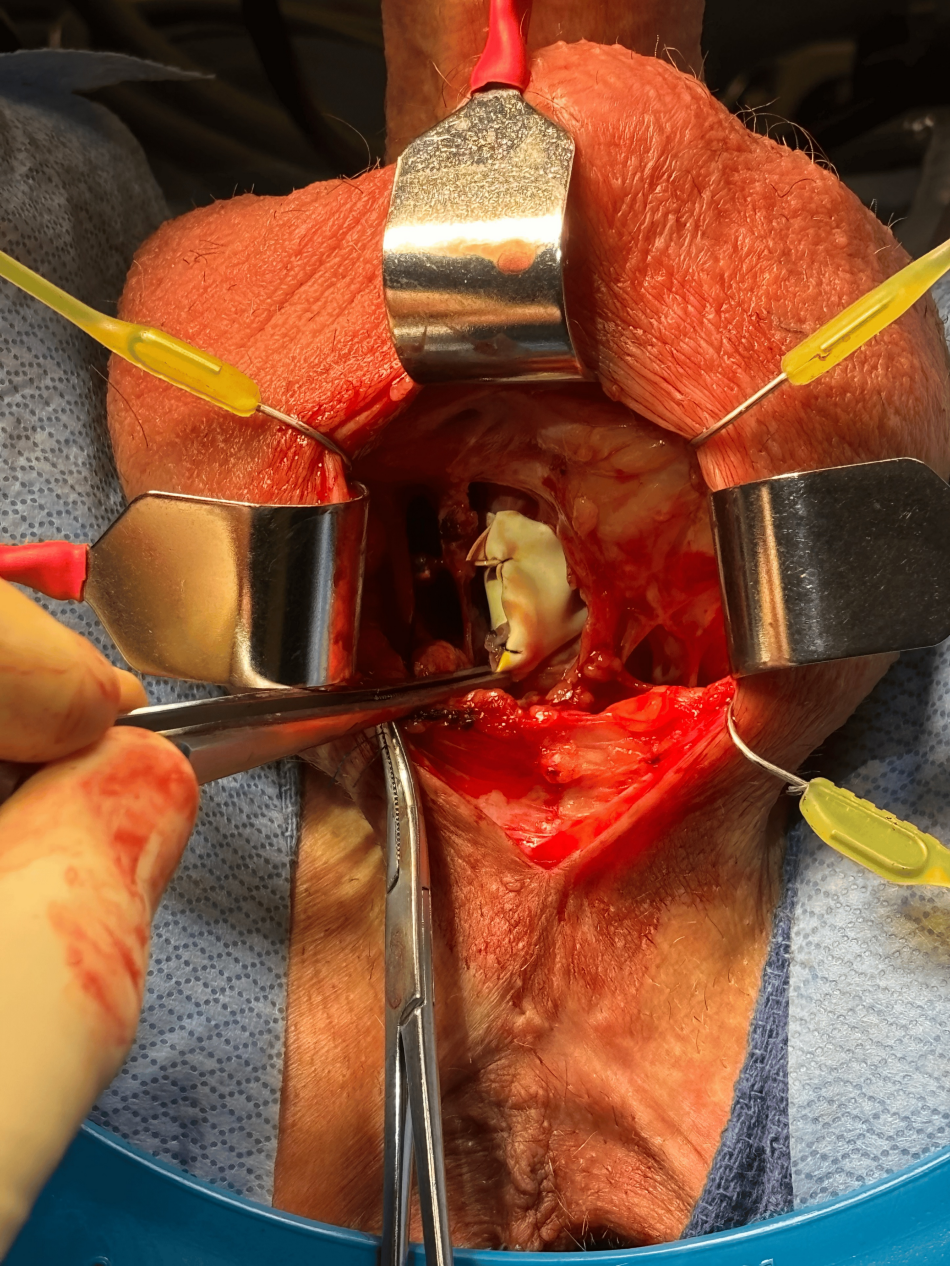
Penrose drain as a spacer around the urethra.

**Figure 2:**
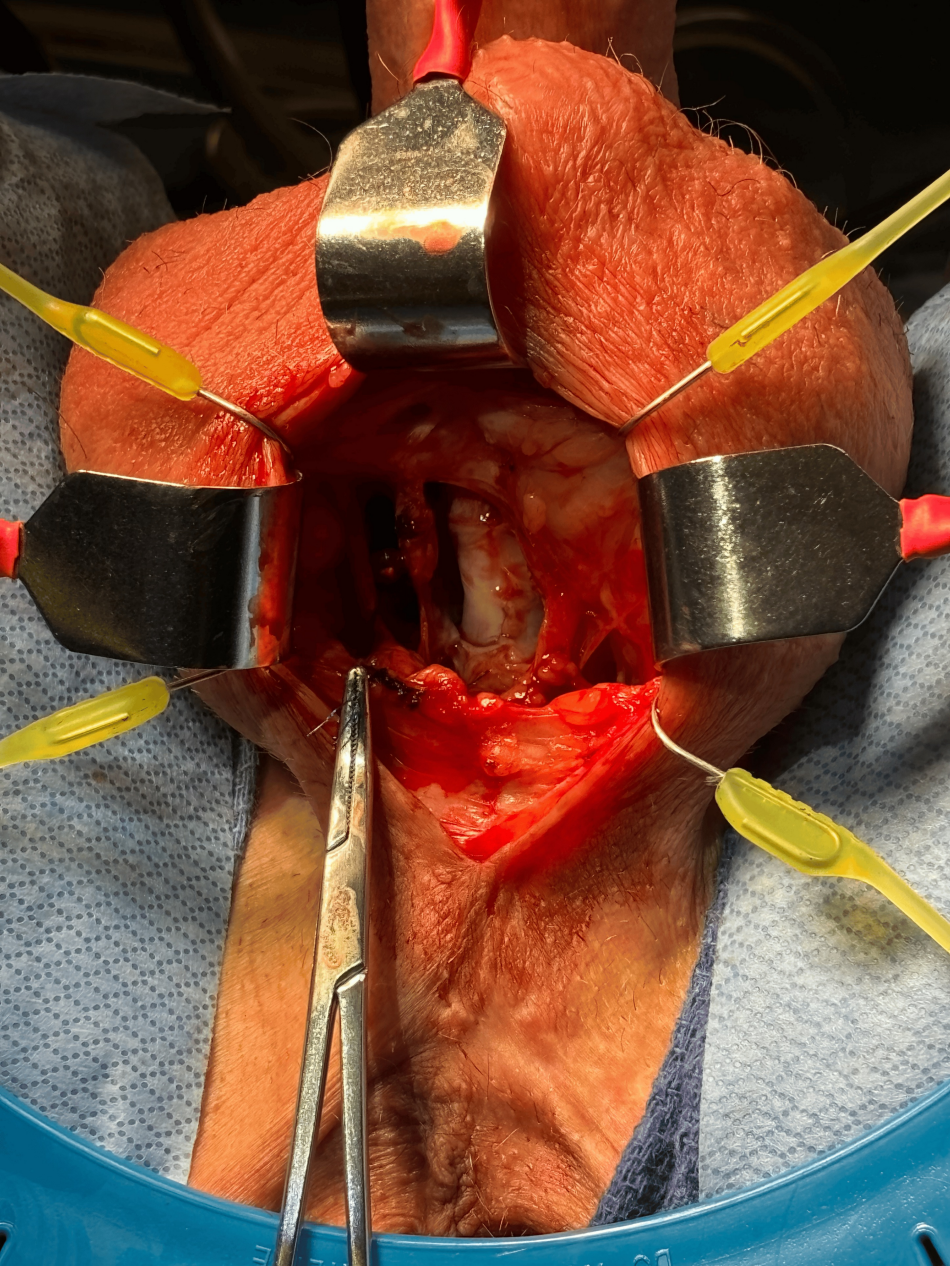
Healthy urethra underneath the Penrose drain.

The patient underwent a transperineal repair of the fistula, incorporating a buccal mucosa graft and gracilis muscle flap to ensure robust tissue healing. Concurrent ileostomy was performed. Due to the presence of the fistula, the AUS cuff was removed to avoid further urethral compromise. During this procedure, a 1” Penrose drain was innovatively used as a temporary spacer (Figure 1), ensuring that no additional urethral dissection would be needed during re-implantation of the AUS cuff. His gastrointestinal tract was confirmed to be intact and ileostomy was reversed about six months post-repair. One month later, cystoscopy confirmed the health of the urethra, with no recurrence of the fistula or erosion and the patient was scheduled for follow-up surgery. At reimplantation of the AUS approximately nine months after the RUF repair, direct inspection after removal of the spacer showed a healthy urethra (Figure 2).

Upon cuff replacement surgery, fluid loss from the balloon reservoir was noted to have occurred. All AUS components were replaced without complications, which included removal of the 1” Penrose drain used as a spacer and subsequent replacement with a new 4 cm AUS cuff. At follow-up, the patient showed no signs of infection or erosion.

## Discussion

This case demonstrates the novel use of a Penrose drain as a urethral spacer after AUS cuff removal. Penrose drains are typically used to prevent fluid accumulation post-surgery; however, their application as a spacer in this scenario offers several advantages. The peri-urethral Penrose drain helps in maintaining the peri-urethral space and minimizing future peri-urethral dissection. The drain helps keep the peri-urethral space open, preventing fibrosis during the healing process. This is particularly important in patients who will undergo future re-implantation of an AUS. In complex re-do AUS surgeries where tissue healing and contraction could compromise urethral dissection, a spacer helps maintain the anatomical space and precludes the need for further dissection, which could further compromise urethral blood supply.

This Penrose drain allows for placement of a urethral catheter during the healing process: This allows for appropriate urethral drainage during the healing process from the complex rectourethral fistula repair. Any prolonged urethral catheter drainage with an AUS cuff in place, whether deactivated or not, carries a risk of urethral erosion. The proposal here is that the Penrose holds the space open, but with less peri-urethral pressure than a deactivated AUS cuff.

One possible advantage is the potential reduction of risk for future sequelae such as secondary AUS failure: This technique may have the potential to lower risk for future AUS cuff-related sequelae. Additional urethral dissection and/or scarring in a radiated patient would increase the risk of erosion. Erosion in a complicated patient like this would likely lead to inability to replace the AUS and may lead to undesirable outcomes such as a need for urinary diversion [5].

The patient’s successful fistula repair, followed by the re-implantation of the AUS nine months later, underscores the efficacy of this technique. The preserved peri-urethral space and healthy urethra confirmed by cystoscopic examination enabled uncomplicated AUS cuff replacement and may reduce the rate of sequelae such as replacement cuff erosion and subsequent urinary diversion. Future studies should compare AUS re-implantation outcomes between patients utilizing Penrose drains as spacers and those without, providing insight into the efficacy of this novel technique for preventing sequelae associated with RUF formation or cuff erosion.

## Conclusions

The use of a Penrose drain as a spacer during AUS removal in cases of RUF repair represents a novel, effective strategy to preserve urethral integrity for future interventions. This case highlights the importance of maintaining the peri-urethral space in complex AUS revision/removal. Future investigations might compare the rates of AUS reimplantation failure in Penrose drain spacer placement against non-placement as a strategy for mitigating potential sequelae such as increased rates of infection, persistent incontinence, and cuff erosion.
